# Health and well-being benefits of spending time in forests: systematic review

**DOI:** 10.1186/s12199-017-0677-9

**Published:** 2017-10-18

**Authors:** Byeongsang Oh, Kyung Ju Lee, Chris Zaslawski, Albert Yeung, David Rosenthal, Linda Larkey, Michael Back

**Affiliations:** 1 Northern Sydney Cancer Centre, Royal North Shore Hospital, Sydney Medical School, Sydney, NSW Australia; 2 School of Life Science, University of Technology, NSW, Australia; 3 Department of Epidemiology and Medical Informatics, Graduate School of Public Health, Korea University, Seoul, South Korea; 4 Harvard Medical School, Boston, MA USA; 5 College of Nursing & Health Innovation, Arizona State University, Phoenix, AZ USA

**Keywords:** Nature, Forest, Health, Wellbeing, Green environment

## Abstract

**Background:**

Numerous studies have reported that spending time in nature is associated with the improvement of various health outcomes and well-being. This review evaluated the physical and psychological benefits of a specific type of exposure to nature, forest therapy.

**Method:**

A literature search was carried out using MEDLINE, PubMed, ScienceDirect, EMBASE, and ProQuest databases and manual searches from inception up to December 2016. Key words: “Forest” or “Shinrin -Yoku” or “Forest bath” AND “Health” or “Wellbeing”. The methodological quality of each randomized controlled trials (RCTs) was assessed according to the Cochrane risk of bias (ROB) tool.

**Results:**

Six RCTs met the inclusion criteria. Participants’ ages ranged from 20 to 79 years. Sample size ranged from 18 to 99. Populations studied varied from young healthy university students to elderly people with chronic disease. Studies reported the positive impact of forest therapy on hypertension (*n* = 2), cardiac and pulmonary function (*n* = 1), immune function (*n* = 2), inflammation (*n* = 3), oxidative stress (*n* = 1), stress (*n* = 1), stress hormone (*n* = 1), anxiety (*n* = 1), depression (*n* = 2), and emotional response (*n* = 3). The quality of all studies included in this review had a high ROB.

**Conclusion:**

Forest therapy may play an important role in health promotion and disease prevention. However, the lack of high-quality studies limits the strength of results, rendering the evidence insufficient to establish clinical practice guidelines for its use. More robust RCTs are warranted.

## Background

There is a growing interest in the health benefits associated with individuals undertaking outdoor activities in a natural environment [1, 2]. Forests and other natural environments are recognized as fundamental health resources and may play a role in disease prevention [3], with one population survey reporting that the average person spends almost 90% of their life indoors [4].

Since the development of the concept of nature as a therapy in the 1990s [5, 6], a number of studies using a variety of methodologies, have been conducted to examine the effect of forest environments on health promotion and well-being. Several recent environmental studies have claimed that a number of medical symptoms related to lifestyle stress can be treated by encouraging individuals to interact with nature [7]. Furthermore, one study revealed an association between positive health outcomes and the amount of exposure an individual has to a green environment [8].

Moreover, research conducted in a very specific natural context, exposure to “forests”, or “forest therapy”, have reported potential benefits in the management of psychological symptoms including anxiety [9], depression [9], mood disorder [10], burnout syndrome [11], lifestyle-related stress [12], and overall quality of life [13]. Studies investigating the effect of forest therapy on physiological well-being have also demonstrated a positive impact on cognitive function [14], immune function [15, 16], blood glucose levels in diabetic patients [17], hypertension [18], cardiovascular disease [19], cancer [20], and pain [21]. Earlier studies conducted with surgical patients suggest that exposure to a green environment is associated with the recovery of illness and even decreased mortality [22–24].

Despite a number of studies providing preliminary evidence linking exposure to a natural, green forest environment and positive psychological and physiological benefits [25–27], most health professionals have failed to recommend nor integrate such therapy for the management or prevention of disease during their medical consultations [24]. As yet, there are no evidence-based clinical practice guidelines for the use of such therapy for healthcare professionals. In previous literature reviews [1], authors have attempted to draw conclusions regarding the therapeutic benefits of forest therapy but doubt remains regarding its efficacy and value [28].

While the theory of forest therapy [5, 6], the belief that taking time out in nature, specifically a forest area, can affect human well-being and heath, was first proposed in Western countries, it was researchers from Japan that instigated studies to evaluate the therapeutic effect of spending time in forests on health and well-being. These Japanese studies influenced further research in Korea, China, and Europe. It was the Japanese who coined the term “shinrin-yoku” to depict activities of recreation and relaxation in a forest. Shinrin-yoku specifically relates to the activity of forest bathing, staying and/or walking in forest, and breathing in the volatile substances released by the trees [17]. Hence, we conducted a further literature review to evaluate the current evidence of spending time in natural green environment, specifically, forest bathing. Our research question is “what, if any, evidence is there that forest bathing has effects on health, and if so, what health indicators show improvement with this exposure?”

## Methods

### Search strategy

A literature search was undertaken using the MEDLINE, PubMed, ScienceDirect, EMBASE, and ProQuest Central databases from inception up to December 2016. The key words used were “Forest” or “Shinrin -Yoku” or “Forest bath” AND “Health” or “Wellbeing”. A manual search of the references of the retrieved articles was also undertaken. Inclusion criteria were publications that reported the effects of forest exposure on health and well-being, tested in the context of a randomized controlled trial (RCT), adult participants, and with the full article published in the English language. Epidemiological studies, case studies, and qualitative and non-human trials were excluded.

### Data extraction

A review template was developed specifying the key information to be extracted from each study and to assess quality as shown in headings in Tables 1 and 2. Two reviewers (BO and KL) independently applied the inclusion and quality assessment criteria. The two reviewers compared results and resolved any disagreement concerning items related to the published articles.

**Table 1:** Characteristics of the trials included in the systematic review

Author Year Publisher	Study design, Location	Sample size, age Study population	Intervention group	Duration of intervention	Control group	Outcome Measurement	Results and conclusion
Shin et al. [1] 2012 Environ Health Prev Med	RCT Korea	*N* = 92 Age (mean 45.26 ± 3.89 years); gender (84 males, 8 females) Chronic alcoholic	*N* = 47 The 9-day forest healing camp 3 days: interaction with Nature 3 days: challenge including mountain climbing and tracking 3 days: self-introspection including mediation and counseling	9 days	*N* = 45 Normal daily routine	Depression measured with Beck Depression Inventory (BDI)	There was a significant difference between two groups (*t* = − 6.27; *p* ≤ 0.001) in favor of forest camp group. Forest environment has a potential effect on depression of chronic alcoholics.
Mao et al. [2] 2012 Journal of Cardiology	RCT China	*N* = 24 Aged from 60 to 75 years Patients with diagnosed essential hypertension. BP, with or without medical control, less than 180/110 mmHg	*N* = 12 Mountain forest Participants walked at an unhurried pace for about 1.5 h, with a 20-min rest during the walk in the morning and afternoon (total 3 h walk/day), They were allowed to do as they wished in the hotel, though avoiding strenuous exercise and any stimulating activities in their hours of relaxation before sleeping.	7 days 7 nights.	N = 12 Urban area Same amount of walking hour intervention at city.	BP with mercury sphygmomanometer Cytokines [interleukin-6 (IL-6) and tumor necrosis factor α (TNF-α) were analyzed with radioimmunoassay kits Cardiovascular disease associated factors: endothelin-1 (ET-1), homocysteine (Hcy), renin, angiotensinogen (AGT), angiotensin II (Ang II), angiotensin II type 1 receptor (AT1) and angiotensin II type 2 receptor (AT2) in sera were measured with enzyme-linked immunoassay Mood status were measured with POMS Air quality, concentration of PM10 (particulate matter considered as mass defined by size cutoff at 10 μm in aerodynamic diameter) was measured by a portable laser dust monitor	Subjects exposed to the forest environment showed a significant reduction in blood pressure in comparison to that of the city group (*p* < 0.05). The serum IL-6 level was significantly reduced in the forest-bathing group compared with its baseline level but not in city group. However, the TNF-α level remained unaltered in both groups during the experiment. The values for the bio-indicators in subjects (ET-1, Hcy, AGT, AT1) exposed to the forest environment were also lower than those in the urban control group (*p* < 0.05). The POMS evaluation showed that subjects had lower scores in the negative subscales, and increased score for vigor in the forest environment group. The air quality in the forest environment was much better than that of the urban area. Forest bathing has therapeutic effects on human hypertension and induces inhibition of the renin–angiotensin system and inflammation, and thus inspiring its preventive efficacy against cardiovascular disorders.
Mao et al. [3] 2012 Biomedical and Environmental Sciences	RCT China	*N* = 20 Mean age 20.79 ± 0.54 years Normal male university students	*N* = 10 Mountain forest Participants walked at an unhurried pace for about 1.5 h, with a 10-min rest during the walk. In the afternoon, after taking lunch in the resting room, the participants walked another area at an unhurried pace for about 1.5 h, with a 10-min rest during the walk.	2 days	*N* = 10 Urban area Same hour walking intervention at city.	Superoxide dismutase (SOD) was examined according to the xanthine oxidase method using a standard assay kit. Lipid peroxidation was evaluated by measuring MDA concentrations Cytokines IL-6, TNF-α, and ET-1 were analyzed with radioimmunoassay kits. Cortisol and testosterone levels in serum were measured with chemiluminescent immunoassay. Lymphocyte assay: To determine lymphocyte subsets, CD5+/CD19+ (B cells), CD3+ (T cells), CD3+/CD4+ (T-helper cells), CD3+/CD8+ (T suppressor cells), and CD3−/CD16+/CD56 + (NK cells), Mood status were measured with POMS	Subjects exposed to the forest environment showed reduced oxidative stress and pro-inflammatory level were as evidenced by decreased MDA, IL-6, and TNF-α levels compared with the urban group (*p* < 0.05). Serum cortisol and testosterone levels were also lower than in the urban group (*p* < 0.05). The concentration of plasma ET-1 was much lower in subjects exposed to the forest environment (*p* < 0.01). The POMS evaluation showed that after exposure to the forest environment, subjects had lower scores in the negative subscales, and the score for vigor was increased (*p* < 0.05). Exposure to the forest environment, even for a short time, may have positive impact on human health.
Lee et al. [4] 2014 European Journal of Integrative Medicine	RCT Korea	*N* = 70 Intervention group mean age: 70.19 ± 4.66 Control group mean age: 71.11 ± 5.80 Elderly femal	*N* = 50 Forest walking	1 h in the morning	*N* = 20 City walking	Arterial stiffness was measured with the cardio-ankle vascular index (CAVI). Pulmonary function was measured with a portable Vitalograph, Copd-6 m: forced expiratory volume in 1 s (FEV1) and forced expiratory volume in 6 s (FEV6)	Forest walking group significantly improved CAVI (*p* < 0.01), FEV1 (*p* < 0.01) and FEV6 (*p* < 0.01). No significant change was observed in the city-walking group. There were significant differences in changes of CAVI (*p* < 0.01), FEV1 (*p* = 0.02), and FEV6 (*p* = 0.04), between the groups. Both systolic and diastolic blood pressure decreased significantly in forest walking group but did not changed in city walking group. No significant side effects were reported.
Sonntag-Öström et al. [5] 2015 Scandinavian Journal of Forest Research	RCT Sweden	*N* = 99 Intervention group mean age: 44.6 (9.1) Control group mean age: 44.5 (8.1) Female (*n* = 85) and male (*n* = 14) patients diagnosed with exhaustion disorder (ED)	*N* = 51 Forest rehabilitation group with subsequent cognitive behavioral rehabilitation (CBR) for all participants	Twice a week for 11 weeks (22 visit in total) Each time 4 h Follow up at 3 months and at the end of the CBR (1 year) in both groups	*N* = 48 Waiting list group with subsequent CBR.	Burnout level were measures with the Shirom Melamed Burnout Questionnaire (SMBQ), Stress with the Perceived Stress Questionnaire (PSQ), Fatigue with the Checklist Individual Strength questionnaire (CIS), Self-esteem with the Self-Concept Questionnaire (SCQ), Anxiety and depression with the Hospital Anxiety and Depression Scale (HAD-S), Mental state was evaluated with the questionnaire asked about the participant’s perceived tenseness (tense/relaxed), fatigue (exhausted/alert), mood (sad/happy), irritability (irritated/harmonious), restlessness (restless/ peaceful) and clear-headedness, Attention capacity was tested using the Necker Cube Pattern Control task (NCPC), Sick leave data	Both groups had enhanced recovery from ED after the 3-month intervention period and at the end of the CBR (1 year). There were no significant differences between the groups in terms of psychological health measures. Mental state was improved, but it showed some seasonal differences. A significant effect on attention capacity was found for single forest visits, but there was no effect found for the rehabilitation period as a whole. The most popular forest environments contained easily accessible, open and bright settings with visible water and/or shelter. Forest rehabilitation did not enhance the recovery from ED compared to the control group, but the participants’ well-being was improved after single forest visits.
Jia et al. [6] 2016 Biomedical and Environmental Sciences	RCT China	*N* = 18 Forest intervention: Age (67–77) Control: Age (61–79) Elderly patients with COPD.	*N* = 10 Forest Walk 90 min in the morning and 90 min in the afternoon (total 3 h) and stayed at hotel	1 day	*N* = 8 Urban 90 min in the morning and 90 min in the afternoon (total 3 h) and stayed at hotel	Cytokine (IFN-γ, IL-6, IL-8, IL-1β, TNF-α) measured with ELISA kits, Relative and absolute number of NK cells (CD56+/CD3−), NK-like (CD56+/CD3−), CD8+ T cells (CD3+/CD8+), perforin and granzyme B expression were measured with flow cytometry, Mood status with POMS	There was a significant decrease of perforin (NK cells, NK-like, CD8+ T cells) (*p* < 0.05) and granzyme B expressions (NK cells, NK-like, CD8+ T cells), accompanied by decreased levels of pro-inflammatory cytokines (IFN-γ, IL-6, IL-8, IL-1β), C-reactive protein (CRP) and stress hormones (cortisol and epinephrine) in the forest group (*p* < 0.05). The scores in the negative subscales of POMS decreased after forest bathing trip (*p* < 0.05. The forest bathing trip has health effect on elderly patients with COPD by reducing inflammation and stress level.

*AGT* angiotensinogen, *AT* angiotensin, *BP* blood pressure, *BDI* Beck Depression Inventory, *CRP* C-reactive protein, *CAVI* cardio-ankle vascular index, *CIS* Checklist Individual Strength questionnaire, *ET-1* endothelin-1, *Hcy* homocysteine, *Lymphocyte subsets* CD3+/CD4+ (T-helper cells), CD3+/CD8+ (T suppressor cells), and CD3−/CD16+/CD56+ (NK cells), *CD* cluster of differentiation, *NK cells* natural killer cells, *IFN-γ* interferon gamma, *IL-6* interleukin-6, IL-8 interleukin-8, IL-1β interleukin-1β, *TNF-α* tumor necrosis factor α, *COPD* chronic obstructive pulmonary disease, *ED* exhaustion disorder, *FEV* forced expiratory volume, *HADS* Hospital Anxiety and Depression Scale, *RCT* randomized controlled trials, *ROS* risk of bias, *MDA* malondialdehyde, *NDI* neck disability index, *NCPC* Necker Cube Pattern Control task, *POMS* profile and mood state questionnaire, *SMBQ* Shirom Melamed Burnout Questionnaire, *PSQ* Perceived stress questionnaire, *SCQ* Self-Concept Questionnaire, *SOD* Superoxide dismutase

**Table 2:**
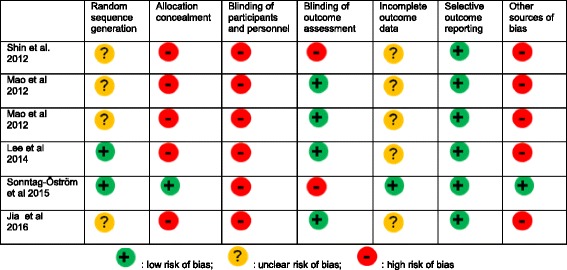
Assessment of risk of bias based on the Cochrane risk of bias tool

### Quality assessment of RCT

The Cochrane risk of bias (ROB) tool (version 5.1.0) [29] was used to evaluate the quality of the RCTs. The ROB assessment tool has seven domains including random sequence generation, allocation concealment, blinding of participants and personnel, blinding of outcome assessment, incomplete outcome data, selective outcome reporting, and other sources of bias [29]. Two reviewers (BO and KL) ascertained the ROB for each domain by indicating high risk, low risk, or unclear risk and resolved disagreements by consensus.

## Results

### Study characteristics

**Fig. 1:**
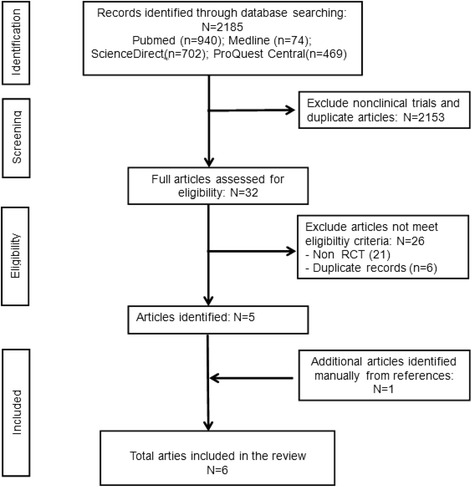
Flowchart of the study selection process

The searches identified 32 potentially relevant articles based on the search terms, of which, 26 articles were excluded as they did not meet the eligibility criteria. Five articles were identified as RCTs including quantitatively measured outcome assessments and were included. An additional RCT was found in a reference list and was included in the review (Fig. 1). Of these 6 RCTs, 3 studies reported both psychological and physiological measures.

Across the 6 studies, the total number of participants was 323. Sample sizes varied from 18 to 99. Three studies had small samples (≤ 60). The other three studies’ sample sizes were all greater than 60 (ranged 61–99). Age of participants ranged from 20 to 79 years. Study population varied from young healthy university students to elderly with chronic disease. Of the 323 participants, 72% (*n* = 233) had diagnosed medical conditions [chronic alcoholic (*n* = 92), hypertension (*n* = 24), exhaustion disorder (*n* = 99), and chronic obstructive pulmonary disease (COPD) (*n* = 18)]. The studies were performed in China (*n* = 3), Korea (*n* = 2), and Sweden (*n* = 1). The duration of forest therapy interventions varied across the studies, ranging from 1 day to 11 weeks. Most forest interventions were conducted with short-term interventions ranging from 1 to 9 days: 1 day (*n* = 2), 2 days (*n* = 1), 7 days (*n* = 1), and 9 days (*n* = 1), but one study was carried out over 11 weeks. Outcome measures varied across the studies using different instruments to measure the effect of forest therapy on physical health and psychological outcomes. Additionally for three studies, several biomarkers were used to measure the effects on physiological outcomes that were dependent on the participants’ medical symptoms. No adverse events were reported. Only one study reported the samples size calculation in relation to the statistical power.

## Physiological response

### Blood pressure

Two studies reported a positive impact on blood pressure (BP). One study conducted in participants with essential hypertension (*n* = 24) reported that participants exposed to the forest environment showed a significant reduction in BP compared to the urban intervention group (*p* < 0.05) [19]. In addition to blood pressure, the values of biomarkers (endothelin-1 (ET-1), homocysteine (Hcy), angiotensinogen (AGT), angiotensin (AT)1) in participants exposed to the forest environment were also lower than those in the urban control group (*p* < 0.05). The second study evaluated the effect of forest walking on elderly women (*n* = 70) and reported that the forest intervention group significantly reduced BP from pre to post forest exposure, while no significant changes were observed in a city walking group [30]. This study did not report the difference of BP between groups.

### Immune function

One study reported a positive effect from forest therapy on immune function. They found significant decreases in the expression of perforin (natural killer cells(NK cells), NK-like, CD8+ T cells) (*p* < 0.05) and granzyme B expressions (NK cells, NK-like, cluster of differentiation (CD)8+ T cells) which were associated with the pathogenesis of chronic obstructive pulmonary disease (COPD) after 1 day spent in a forest environment, while there was no differences detected in the urban intervention group [31].

### Inflammation

Three RCTs reported favorable changes of pro-inflammatory cytokines and C-reactive protein (CRP) [19, 31, 32] in the forest intervention group. Two of these studies evaluated the effect of forest intervention on inflammation compared to an urban intervention. A study conducted with essential hypertension patients showed that the interleukin-6 (IL-6) level was significantly reduced in the forest pre-post intervention group (*p* < 0.05) but not in urban control group. Further, it reported that the tumor necrosis factor α (TNF-α) level did not change in either group [19]. However, a study conducted with young university students by the same investigator reported that both IL-6 and TNF-α levels were significantly lower in the forest intervention group compared to the urban intervention group (*p* < 0.05) [32]. Another recent RCT conducted with COPD patients reported that pro-inflammatory cytokines (IFN-γ, IL-6, IL-8, IL-1β) and CRP were lower in the forest group compared to the urban group (*p* < 0.05) [31].

### Oxidative stress and antioxidant

One study evaluated the effect of forest therapy on oxidative stress and antioxidant levels on young college students [32]. Oxidative stress and antioxidant activity were evaluated using the biomarkers of malondialdehyde (MDA) and superoxide dismutase (SOD), respectively. Results showed significant differences in MDA in the forest intervention group compared to the urban group (*p* < 0.001) but not for SOD.

### Cardiac and pulmonary function

One study examined the effect of forest intervention on arterial stiffness and pulmonary function in elderly women [30]. Arterial stiffness and pulmonary function were measured with cardio-ankle vascular index (CAVI) that measures arterial stiffness of the artery from the heart to the ankles and Vitalograph, Copd-6 m respectively. Results of this study showed that there were significant differences in changes of CAVI (*p* < 0.01), forced expiratory volume in 1 s (forced expiratory volume (FEV1)) (*p* = 0.02), and forced expiratory volume in 6 s (FEV6) (*p* = 0.04) in the forest walking group compared to the urban walking group.

### Stress and stress hormone

One study evaluated stress levels in women diagnosed with exhaustion disorder (ED). Their stress level was measured with the Perceived Stress Questionnaire (PSQ). This study reported that both the forest intervention and control (waiting list) group decreased stress after a 3-month intervention period but there was no difference between the groups [33]. Another study evaluated the effect of forest intervention on the stress hormone serum cortisol and reported that cortisol levels were significantly lower in the forest intervention group compared to the urban intervention group (*p* < 0.01) [32].

## Psychological outcomes

### Anxiety and depression

Two studies measured the effect of the forest environment intervention on anxiety and depression. One study involved alcoholics which measured depression with the Beck Depression Inventory (BDI) and reported significant improvement of depression for the forest intervention group compared to the usual care control group (*p* < 0.001) [10]. In contrast, another study evaluated the outcome of anxiety and depression with the Hospital Anxiety and Depression Scale (HADS) in women diagnosed with exhaustion disorder (ED) and reported that both the forest intervention and the wait-list control groups improved their anxiety and depression scores after 3-month intervention period with no significant difference between the groups [33].

### Mood

Three studies measured psychological responses with profile and mood state questionnaire (POMS) which is used to measure mood disorders [19, 31, 32]. Study populations were diagnosed with hypertension [19] and COPD [31] and included healthy university students [32]. Studies reported that the forest therapy intervention group had significantly lower scores in the negative subscales (tension-anxiety, depression, anger-hostility, fatigue, and confusion) [19, 31, 32] and increased vigor (*p* < 0.05) [19, 32].

### Assessment of risk of bias

In the assessment of random sequence generation, two RCTs had low ROB and four had unclear ROB. Two studies reported the method of random sequence generation while four studies did not describe the process. Five RCTs had high ROB for allocation concealment due to lack of reporting the allocation concealment process. Only one study described in detail the method of allocation concealment. All studies had a high ROB in the blinding of participants and personnel. The blinding of participants and study personnel is one of the main limitations in the forest intervention study. Two RCTs had high ROB and four RCTs had low ROB in blinding of outcome assessment. Two studies measured outcome with self-reported questionnaires. These can be influenced by lack of blinding, whereas four RCTs measured outcomes with biomarkers in addition to the self-reported questionnaires to minimize the ROB.

Five RCTs had unclear ROB in incomplete outcome data due to the lack of reporting missing outcome data during their data analysis. One RCT had low ROB, due to the reporting of details of missing outcome data including the imputation of such missing data, dropout rate, and statistical power. All RCTs had low ROB in selective reporting. Six studies reported on the aim of study, method of outcome assessment, and results of all outcomes. In the assessment of other sources of bias, three RCTs had small sample size (< 50) and five RCTs had a short duration of forest intervention (< 10 days).

## Discussion

There continues to be conjecture about the value that exposure to nature plays in human health and disease. Numerous case studies and epidemiological and observational studies conducted with forest intervention reported positive health and well-being outcomes among the participants who spent time in a forest, and some benefit was shown to be derived even with simply viewing natural environments. Previous literature reviews attempted to synthesize the results of case studies and epidemiological and observational studies to demonstrate the positive effect of spending time in forest rather than providing evidence based on RCTs. To our knowledge, to date, there has been no systemic literature review conducted to assess the evidence of health and well-being benefits of forest therapy based on RCTs. Hence, this present review builds on the previous work and goes further by including RCTs in the evaluation of the evidence for the physiological and psychological health and well-being benefits of forest therapy.

This present review found that six RCTs reported promising therapeutic benefits of forest exposure on several physical and psychological conditions including hypertension, cardiac and pulmonary function, immune function, inflammation, oxidative stress, stress, stress hormone, anxiety, depression, and emotional response, although outcomes of anxiety and depression had mixed results and some inflammatory biomarkers showed null results. These data show a consistent trend in a broad range of health outcomes, suggesting potential for forest bathing to improve physiological and psychological health in healthy and health-compromised individuals, but these results are drawn mostly from studies with strong to moderate design weaknesses. All studies included in this review had a high ROB (Fig. 1). Two of the studies only reported the differences pre and post intervention within each group, and even though they found significant improvements within the forest therapy group, they failed to report whether there was any difference between groups, thus not testing the significance of between-group differences expected of an rigorously evaluated RCT. Of the six RCTs, five evaluated only the immediate or short-term effect of forest intervention [1 day (*n* = 2), 2 days (*n* = 1), 7 days (*n* = 1), 9 days (n = 1)] without long-term follow-up. Only one RCT examined the effect of 11 weeks of forest intervention. Furthermore, five studies were conducted with small sample sizes and thus failed to meet statistical power required to detect significant therapeutic effects, if any. None of the studies were performed with blinding of subjects. Moreover, none of the studies controlled for potential bias or confounding factors (social interaction of subject, physical activities, and forest environmental factors) during the data analysis thus limiting conclusions which may be drawn regarding the true therapeutic effect of forests. Nonetheless, the present review findings are consistent with previous reviews that suggest benefits; however, the current findings need to be evaluated cautiously due to the majority of the studies having risk of bias, low sample sizes, and lack of control for participant expectation effects [34, 35].

The present review did not find convincing evidence of the benefits of forest therapy due to the lack of high-quality studies. However, we cannot disregard the potential impact of nature in the form of forest therapy on health and disease. The concept of human health and longevity and its relationship with the natural environment has a long history [36]. Several studies suggest that exposure to a green environment is associated with a positive impact on physical and psychological well-being including recovery from illness and even decrease mortality [23, 37, 38]. A recent study also revealed that therapeutic benefits from nature and more specifically a green environment may be dose-dependent [8]. It appears likely that the therapeutic benefit of forest therapy is multi-factorial. It may be induced by the complex ecosystem as a whole such as the green scenery, fresh air, sunlight, clean water, rocks, soil, soothing sounds of streams, waterfalls, birds, and natural aromas of trees, plants, and flowers [39].

Taking into account the complexity of existing research on the therapeutic benefits of nature, researchers should consider the following suggestions to improve the rigor of future studies.

First, evaluation of both short-term intervention (1 week) and long-term intervention (12 weeks) duration is recommended, with multiple follow-ups in the post intervention phase (3 months, 6 months, and 12 months). The dose-response relationship should be examined by varying length (e.g., 30 versus 60 versus 90 min), frequency (e.g., weekly versus biweekly versus every 4 weeks), and intensity of intervention, as measured by a physical activity intensity scale.

Next, the lack of participant blinding in most of these forest intervention studies could be remedied. When the study design is a forest versus city outdoor exposure comparison, having participants believe they are involved in a “walking” study would blind the urban vs. forest walkers to the expected outcome. There may be a benefit in designing a study that also controls for the outdoor activity, for example a three-arm design (forest versus urban walking versus either a waitlist group or an indoor reading group). Adequate sample size is recommended to detect statistical and clinical significance; future studies should include power calculations for sample size selection. Moreover, it is recommended that future studies utilize both quantitative and qualitative approaches that will capture the complexity of the forest environment effect. Measurement of disease-specific biomarkers (e.g., cancer makers for the oncology patient) and overall well-being biomarkers (e.g., immune function, cytokines, and DNA damage) may provide objective information on the physiological and psychological effects of forest intervention. Finally, a cost-benefit analysis of forest intervention should be conducted in order to support the possible implementation of forest therapy from a health economics perspective.

## Conclusion

The objective in this review was to evaluate the health and well-being benefits of a specific type of exposure to nature, forest therapy based on RCTs. In conclusion, strong evidence of the benefits of forest environment on health and well-being has yet to be confirmed. The findings of this review support the premise that exposure to a forest environment may provide benefits. However, the evidence is insufficient due to methodological design flaws. Future research studies with more robust design is warranted. While the beneficial effects of forest therapy require further investigation, given that the intervention has low risk of adverse effects and the likelihood that such a recommendation would also increase outdoor activity, policy makers and health professionals should consider recommending forest therapy to their patients and the general public.

## Abbreviations

AGT: Angiotensinogen
AT: Angiotensin
BDI: Beck Depression Inventory
BP: Blood pressure
CAVI: Cardio-ankle vascular index
CD: Cluster of differentiation
CIS: Checklist Individual Strength questionnaire
COPD: Chronic obstructive pulmonary disease
CRP: C-reactive protein
ED: Exhaustion disorder
ET-1: Endothelin-1
FEV: Forced expiratory volume
HADS: Hospital Anxiety and Depression Scale
Hcy: Homocysteine
IFN-γ: Interferon gamma
IL-6: Interleukin-6
IL-8: Interleukin-8
IL-1β: Interleukin-1β
Lymphocyte subsets: CD3+/CD4+ (T-helper cells), CD3+/CD8+ (T suppressor cells), and CD3−/CD16+/CD56+ (NK cells),
MDA: Malondialdehyde
NCPC: Necker Cube Pattern Control task
NDI: Neck disability index
NK cells: Natural killer cells
POMS: Profile and mood state questionnaire
PSQ: Perceived Stress Questionnaire
RCT: Randomized controlled trials
ROS: Risk of bias
SCQ: Self-Concept Questionnaire
SMBQ: Shirom Melamed Burnout Questionnaire
SOD: Superoxide dismutase
TNF-α: Tumor necrosis factor α
